# 16-Hydroxycleroda-3,13-Dien-15,16-Olide Induces Apoptosis in Human Bladder Cancer Cells through Cell Cycle Arrest, Mitochondria ROS Overproduction, and Inactivation of EGFR-Related Signalling Pathways

**DOI:** 10.3390/molecules25173958

**Published:** 2020-08-30

**Authors:** Yu-Chi Chen, Po-Yu Wang, Bu-Miin Huang, Yu-Jen Chen, Wei-Chang Lee, Yung-Chia Chen

**Affiliations:** 1 Department of Urology, E-Da Cancer Hospital, Kaohsiung 824410, Taiwan; yuchichen1978@gmail.com; 2 School of Medicine, I-Shou University, Kaohsiung 824410, Taiwan; 3 Department of Paediatric Emergency, Changhua Christian Children Hospital, Changhua 500209, Taiwan; dama0115@yahoo.com.tw; 4 Department of Anatomy, College of Medicine, National Cheng Kung University, Tainan 701401, Taiwan; bumiin@ncku.edu.tw; 5 Resen Biomedical Informatics, Inc., Taipei 100043, Taiwan; anemone.c@gmail.com; 6 Graduate Institute of Medicine, College of Medicine, Kaohsiung Medical University, Kaohsiung 807378, Taiwan; weichanglee7202@gmail.com; 7 Department of Anatomy, School of Medicine, College of Medicine, Kaohsiung Medical University, Kaohsiung 807378, Taiwan

**Keywords:** clerodane diterpene, apoptosis, bladder cancer, epidermal growth factor receptor, cell cycle

## Abstract

A clerodane diterpene compound 16-hydroxycleroda-3,13-dien-15,16-olide (CD) is considered a therapeutic agent with pharmacological activities. The present study investigated the mechanisms of CD-induced apoptosis in T24 human bladder cancer cells. CD inhibited cell proliferation in a concentration and time-dependent manner. CD-induced overproduction of reactive oxygen species and reduced mitochondrial membrane potential, associated with reduced expression of Bcl-2 and increased levels of cytosolic cytochrome c, cleaved PARP-1 and caspase-3. In addition, CD treatment led to cell cycle arrest at the G0/G1 phase and inhibited expression of cyclin D1 and cyclin-dependent kinases 2 and 4 and led to increased levels of p21, p27Kip1 and p53. All of these events were accompanied with a reduction of pEGFR, pMEK1/2, pERK1/2, pAkt, pmTOR, pP70S6K1, HIF-1α, c-Myc and VEGF. RNAseq-based analysis revealed that CD-induced cell death was characterised by an increased expression of stress and apoptotic-related genes as well as inhibition of the cell cycle-related genes. In summary, CD induces apoptosis in T24 bladder cancer cells through targeting multiple intracellular signaling pathways as a result of oxidative stress and cell cycle arrest.

## 1. Introduction

16-hydroxycleroda-3, 13-dien- 16, 15-olide (CD) is one of the major active compounds of clerodane diterpenes and has been found to exhibit numerous pharmacological functions, such as anti-lipogenic [1], anti-leishmanial [2], anti-fungal [3], anti-inflammation [4], and anti-cancer activities [5]. *Polyalthia longifolia* is a tall evergreen tree native to Pakistan, India, and Sri Lanka that belongs to the Annonaceae family, also known as “Ashoka” [6]. Phytochemical investigation shows that different parts of the plant contain several different compounds, including clerodane and related diterpenes, triterpenes, alkaloids, and flavonoids [6]. CD has been tested in several cancer cell lines and presents anti-tumour and anti-cancer activities [5,7,8]. However, the anti-tumour effects of CD on human bladder cancer (BC) cells remain unknown.

BC, also known as bladder urothelial carcinoma, is the most common malignancy of the genitourinary tract, with an incidence rate of 350,000–380,000 cases reported per year worldwide, and accounts for 2–3% of the worldwide cancer burden [9]. BC is the fourth most common cancer in America [9] and the ninth most common cancer in Taiwanese males, with an age-standardised rate of 8.70 per 100,000 people in 2012 [10]. The current standard treatment for patients with low to moderate grade BC is intravesical Bacillus Calmette-Guérin instillation after scraping the lesion [11,12]. In addition, combination chemotherapy regimens, such as methotrexate, vinblastine, adriamycin, and cisplatin or gemcitabine plus cisplatin, are commonly used to treat metastatic BC [13]. However, the median survival for recurrent or metastatic BC is 12–15 months with advanced chemotherapy, and there is no approved second-line therapy [13].

Overexpression of epidermal growth factor receptor (EGFR) is common in solid tumours, including breast, lung, prostate, and bladder cancer [14]. The EGFR is a transmembrane protein which acts as a receptor to direct a series of crucial events of proliferation, migration, differentiation, and apoptosis [15]. Two important and major signaling routes in EGFR are Ras-Raf-mitogen activated protein kinase (MAPK) and phosphatidylinositol 3-kinase regulatory subunit alpha (PI3K)-Akt [15]. Constitutive activation of EGFR and its downstream signals results in uncontrolled alteration of gene activity and tumour proliferation [14,15]. The present study aims to determine the mechanisms of CD-induced cell death in the T24 human BC cell line by assessing apoptotic signals, cell cycle distribution, and EGFR-related signalling pathways.

## 2. Results

### 2.1. CD Induces Apoptosis in T24 BC Cells

**Figure 1:**
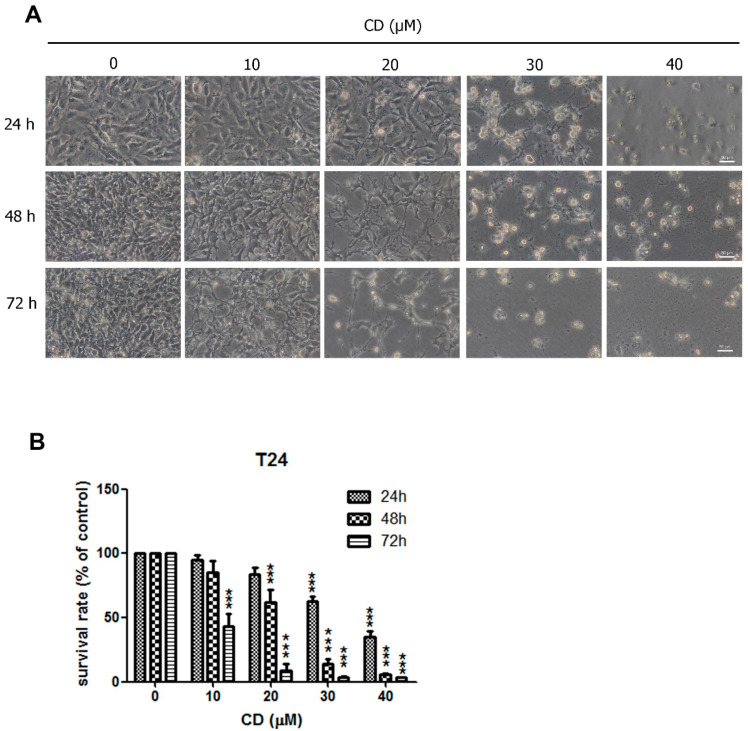
Cytotoxicity induced by CD in T24 BC cells. Cells were treated with different concentrations of CD for 24, 48, and 72 h. (**A**) Cellular morphological changes after treatment with CD. Scale bar = 50 μm. (**B**) Survival rates were examined via MTT assays at 24, 48, and 72 h. *** *p* < 0.001 as compared with control group (0 µM) in the same time period. Bar graphs show normalised values as percentages relative to the control. Data are expressed as mean ± SD of three independent experiments.

To test the cytotoxicity of CD on T24 human BC cells, we used an MTT assay to assess cell activity. Treatment with CD in the micromolar range led to cell death in a time- and concentration-dependent manner (Figure 1). Phase-contrast microscopy revealed that treatment with CD caused cell shrinkage and led to the formation of apoptotic vacuoles (Figure 1A). After long periods of CD exposure (24, 48, and 72 h), BC cells were no longer adherent at concentrations of 30 and 40 µM. Cell damage was seen at 72 and 48 h after treatment with 10 μM and 20 μM CD, respectively (Figure 1B).

**Figure 2:**
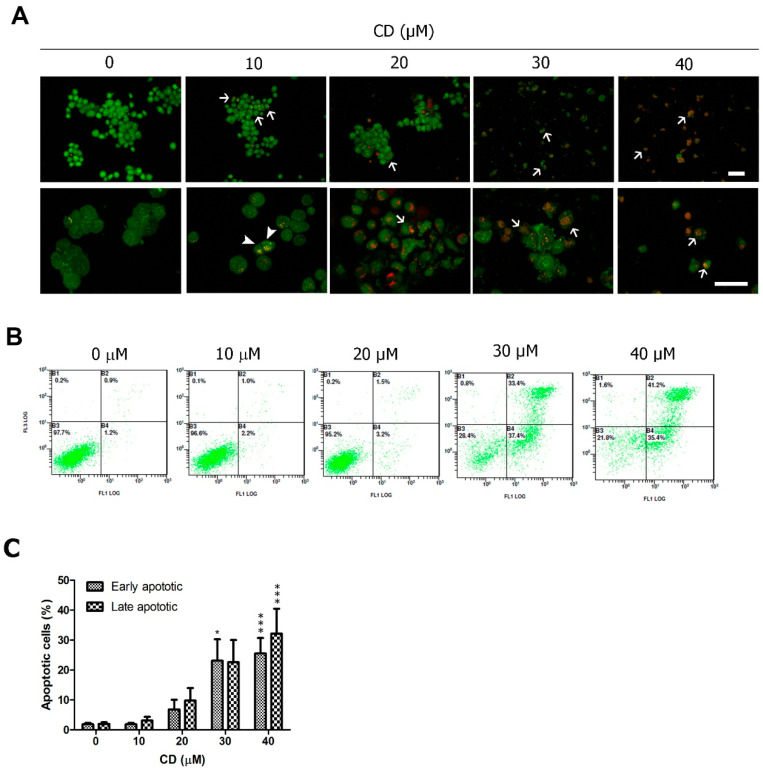
Apoptosis induced by CD in T24 BC cells. (**A**) AO/EB double staining with arrows indicating early apoptotic cells that have fragmented bright green chromatin and late apoptotic cells with a nucleus with fragmented orange chromatin (arrowhead). The upper row is 200× and the lower row is 400× magnification. Scale bar = 50 µm. (**B**) Flow cytometry analysis of annexin V-PI double staining. (**C**) Quantification of apoptotic populations. * *p* < 0.05, *** *p* < 0.001 as compared with the control group (0 µM). Data are expressed as mean ± SD of three independent experiments.

To investigate whether CD-induced cell death is associated with apoptosis, we used AO/EB double staining and the annexin V-PI assay for visualisation and quantification of apoptosis. CD-induced cell shrinkage and apoptosis, and necrosis was rare after 24 h of treatment (Figure 2A). Compared with the control group, CD-treated T24 cells had high levels of early and late apoptotic cells, but lower levels of necrotic cells (<2%) after 24 h of treatment (Figure 2B).

### 2.2. CD Suppresses MMP and Triggers ROS Production

**Figure 3:**
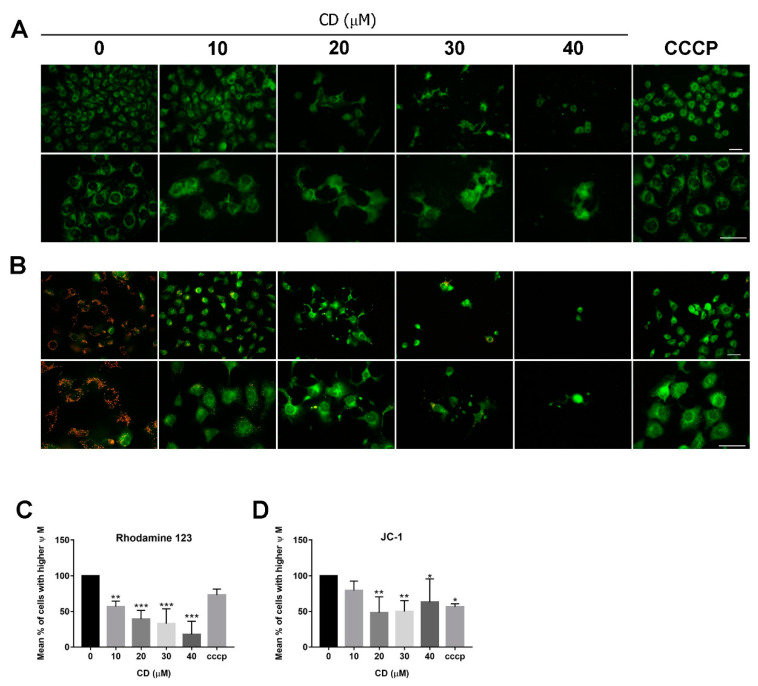
MMP is decreased after treatment with CD in T24 BC cells. Fluorescence staining of mitochondria by (**A**) Rh123 and (**B**) JC-1 at 200× and 400× magnifications. Scale bar = 50 µm. The representative photos were taken from one of the three independent experiments. Flow cytometry analysis of MMP by (**C**) Rh123 (**D**) JC-1 staining. Carbonyl cyanide 3-chlorophenylhydrazone (CCCP) was used as a positive control for confirming the successful inhibition of MMP. Bar graphs show values normalised as percentages relative to the control. Data are expressed as mean ± SD of three (Rh123) and four (JC-1) independent experiments. * *p* < 0.05, ** *p* < 0.01, *** *p* < 0.001 as compared with the control group (0 µM).

To examine whether CD-induced cell death involves mitochondria-dependent apoptotic mechanisms, the fluorescent dyes Rh123 and JC-1 were used to measure the MMP. As shown in Figure 3A, vehicle-treated BC cells exhibit strong green fluorescence in the mitochondria, whereas treatment with CD eliminated Rh123 staining starting at concentrations of 10 μM (Figure 3A). As shown in Figure 3B, there are reduced JC-1 aggregates and monomers in CD-treated cells, especially at 20–40 µM (Figure 3B). We also analysed the MMP using flow cytometry and observed decreased fluorescence intensity after treatment with CD (Figure 3C,D). In summary, compared with the vehicle-treated control, both Rh123 and JC-1 staining in T24 cells were reduced.

**Figure 4:**
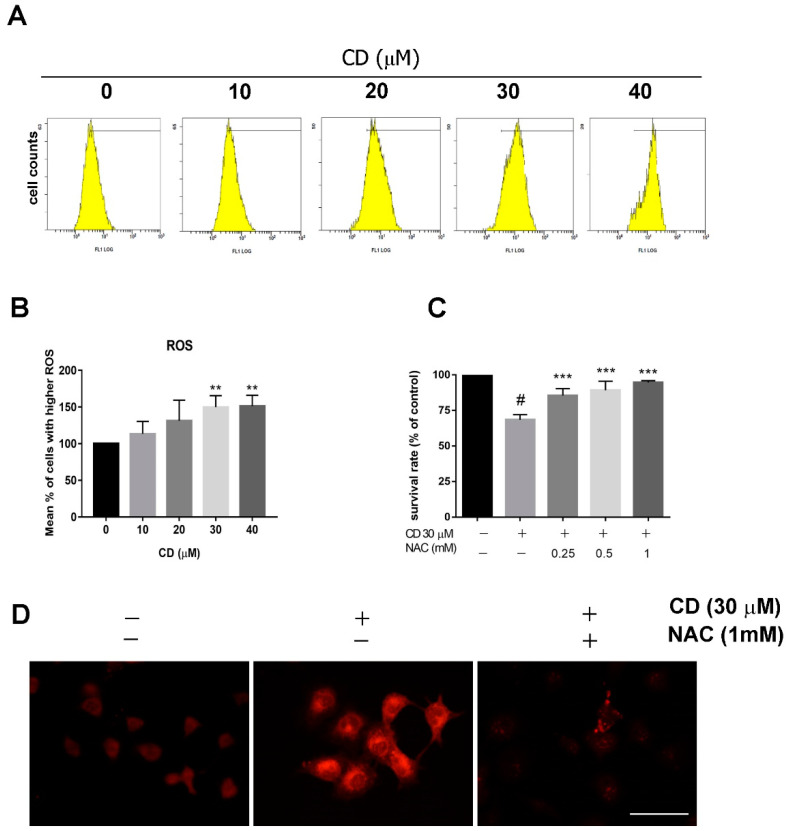
Reactive oxygen species (ROS) overproduction after treatment of T24 BC cells with CD. (**A**) Flow cytometry analysis using a ROS-specific dye. (**B**) Quantification of ROS staining via flow cytometry. (**C**,**D**) Cells were pre-treated with the antioxidant agent N-acetyl cysteine (NAC) for 30 min and then incubated with CD for additional (**C**) 24 h or (**D**) 8 h. MTT assays (**C**) were performed to determine the survival rates. (**D**) MitoSOX fluorescence dye was used to detect mitochondrial ROS. Scale bar = 50 µm. Bar graphs show values normalised as percentages relative to the control. Data are expressed as mean ± SD of four independent experiments. In (**B**), ** *p* < 0.01 compared with control group (0 µM). In (**C**), # *p* < 0.05 compared with control group; *** *p* < 0.001 compared with CD group.

The loss of MMP leads to an energy imbalance and triggers ROS production. We used the fluorescent dye DCFH-DA as an indicator of peroxide and superoxide accumulation. After treatment with CD, we observed a concentration-dependent increase in ROS production (Figure 4A,B). Quantitative analysis showed that treatment with 30 and 40 μM of CD significantly elevated ROS production to 1.5 times basal levels (Figure 4B). Pre-treatment with the antioxidant reagent N-acetyl cysteine (NAC, 0.25–1 mM) reversed CD-induced cytotoxicity in a concentration-dependent manner (Figure 4C). CD-induced mitochondria ROS production can be observed immediately 8 h after treatment (Figure 4D) and the effect can be blocked by NAC.

### 2.3. CD Triggers the Expression of Pro-Apoptotic Proteins and Inhibits Anti-Apoptotic Proteins

**Figure 5:**
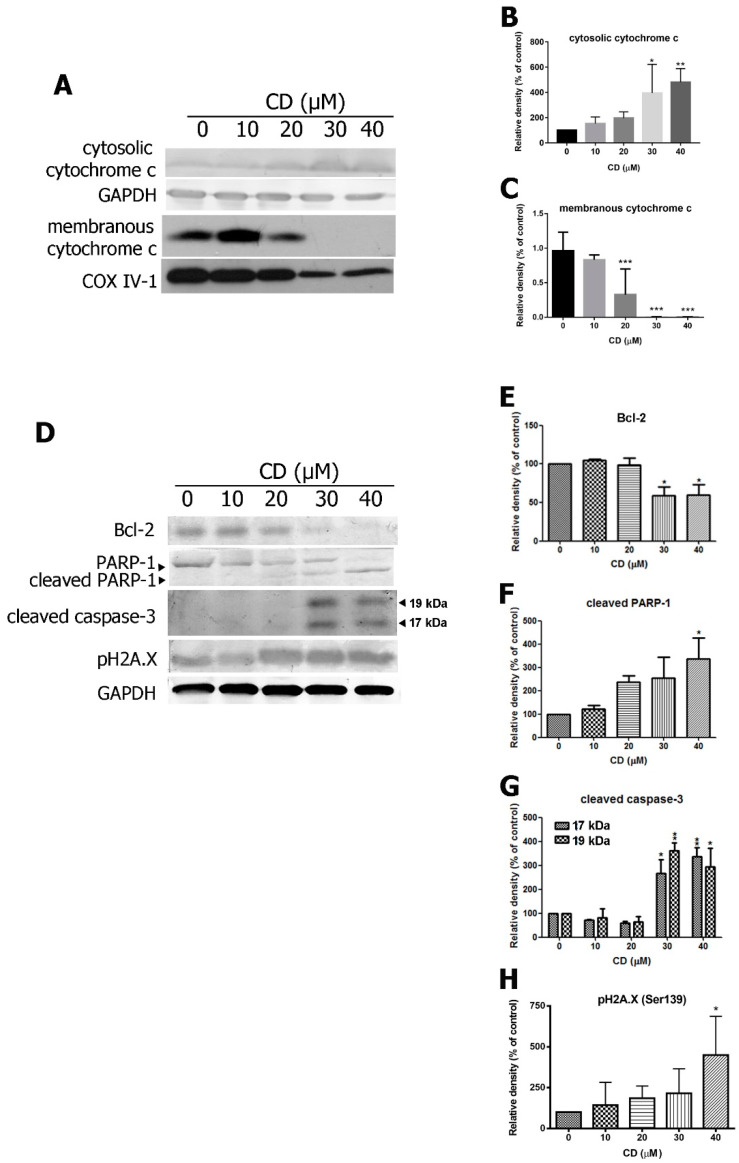
Effects of CD on pro-apoptotic and anti-apoptotic proteins in T24 BC cells. (**A**) The expression of cytochrome c in the cytosolic fraction and membrane fractions. Glyceraldehyde 3 phosphate dehydrogenase (GAPDH) and cytochrome oxidase subunit IV isoform 1 (COX IV-1) were used as internal controls. (**B**,**C**) Quantifications of protein expression. (**D**) The expression of Bcl-2, cleaved PARP-1, 17 kDa, and 19 kDa subunits of cleavage caspase-3 and pH2A.X. (**E**–**H**) Quantifications of the protein expression. Bar graphs show normalised values as percentages relative to the control. Data are expressed as mean ± SD of three to five independent experiments. * *p* < 0.05, ** *p* < 0.01, *** *p* < 0.001 compared with the control group (0 µM).

CD treatment led to increased cytochrome c release from mitochondria to the cytosol (Figure 5A–C). Therefore, investigated downstream signalling molecules in the caspase-related pathway by western and found that treatment with CD enhanced expression of cleaved caspase-3 (17 and 19 kDa subunits), cleaved PARP-1 and pH2A.X (Figure 5D–H). CD treatment also inhibited Bcl-2 anti-apoptotic protein expression. These results suggest that CD induces mitochondrial-dependent apoptosis in BC cells through activation of the caspase-3 dependent pathway.

### 2.4. Effects of CD on Cell Cycle Progression, Cyclins and Cyclin-Dependent Kinases (CDKs)

**Figure 6:**
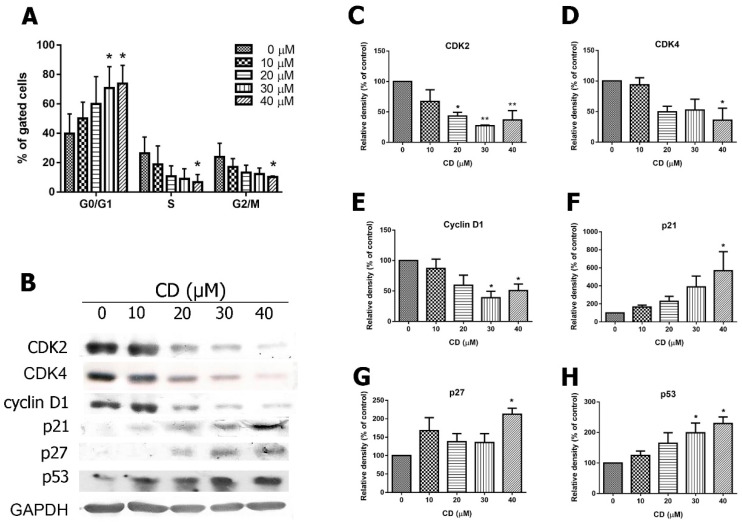
Effects of CD on cell cycle distribution and cell cycle-related protein expression. Cells were treated with the indicated concentrations of CD for 24 h. (**A**) Quantification data of flow cytometry staining with PI. (**B**) Protein expression of p21, p27Kip1, p53, CDK2, CDK4, and cyclin D1. (**C**–**H**) Quantification results of each protein. Bar graphs show values normalised as percentages relative to the control. Data are expressed as mean ± SD of three to four independent experiments. * *p* < 0.05, ** *p* < 0.01 as compared with the control group (0 µM).

To determine whether CD inhibits proliferation, we assessed cell cycle markers in CD-treated T24 cells. Compared with the control (0 µM CD, 39.8% of cells in G0/G1), CD treatment led to a cell cycle arrest of T24 cells (50.1%, 59.95%, 70.9%, and 73.9% of cells in arrest at 10, 20, 30, and 40 μM concentrations of CD, respectively, Figure 6A) in the G0/G1 phase after 24 h of treatment. This was associated with a decrease of the number of cells in the S and G2/M phases of the cell cycle (Figure 6A). Accordingly, the G1 phase regulatory proteins, including cyclin D1, CDK2, CDK4, p21, p27Kip1, and p53 were analysed using western blot. CD treatment reduced protein levels of CDK2, CDK4, and cyclin D1, but increased levels of CDK inhibitors, p21 and p27Kip1, in a concentration-dependent manner after 24 h of treatment (Figure 6B–G). The protein p53 was also elevated via CD treatment (Figure 6H).

### 2.5. CD Modulates the Epidermal Growth Factor Receptor-Mediated Signalling Pathway

**Figure 7:**
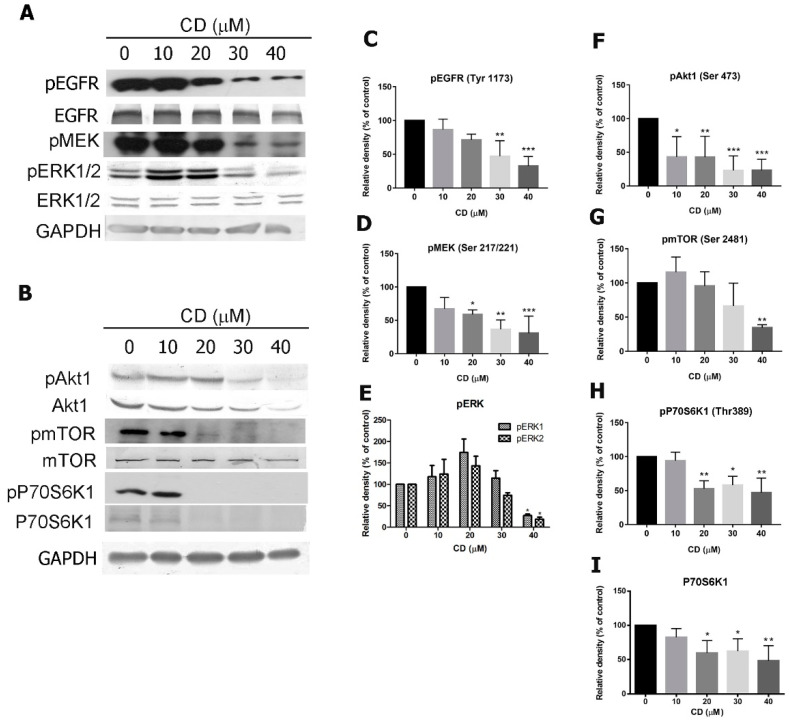
Effects of CD on oncogenic signal molecules. (**A**) The protein expression of pEGFR, EGFR, pMEK, pERK1/2, and ERK1/2. (**B**) The protein expression of pAkt1, Akt1, pmTOR, mTOR, pP70S6K1, and P70S6K1. GAPDH was used as an internal control. (**C**–**I**) Quantification results of each protein. Bar graphs show values normalized as percentages relative to the control. Data are expressed as mean ± SD of three to four independent experiments. * *p* < 0.05, ** *p* < 0.01, *** *p* < 0.001 as compared with the control group (0 µM).

To determine whether CD induces apoptosis by modulation of the EGFR pathway, we measured the expression of pEGFR (Tyr1173), pAkt1 (Ser473), pmTOR (Ser2481), pMEK (Ser 217/221), pERK1/2 (Thr202/Tyr204), and pP70S6K1 (Thr 389) as well as the corresponding total protein levels of each protein. After treatment with varying concentrations of CD (0–40 μM) for 24 h, there was reduced expression of pEGFR (Figure 7A,C), pMEK (Figure 7A,D), pERK1/2 (Figure 7A,E), pAkt1 (Figure 7B,F), pmTOR (Figure 7B,G), pP70S6K1 (Figure 7B,H) and P70S6K1 (Figure 7B,I) in comparison to the control (0 µM) group.

**Figure 8:**
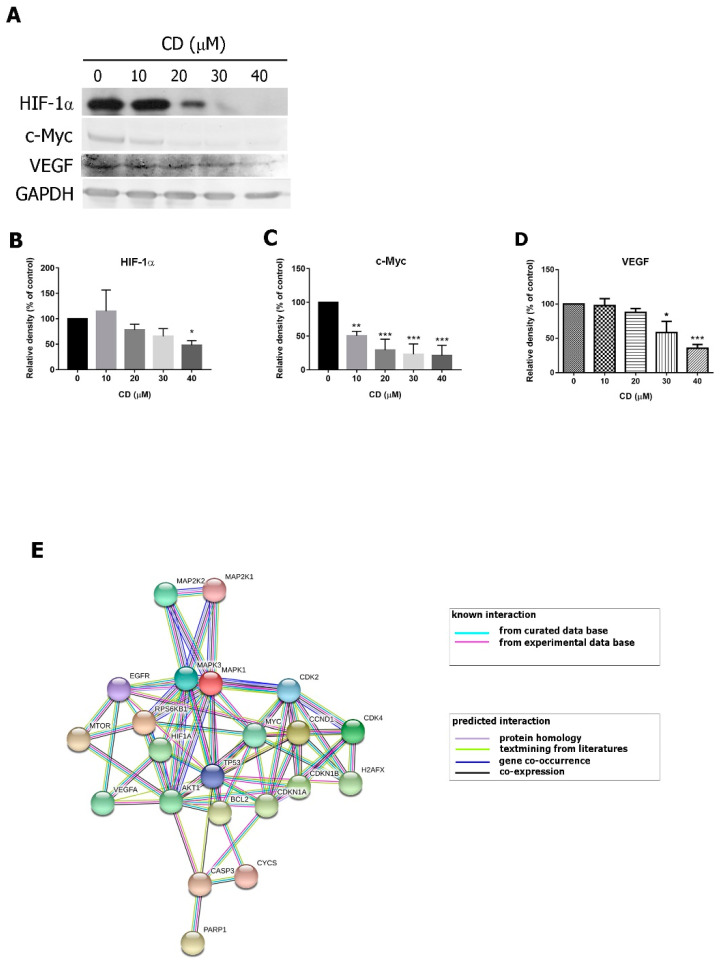
The effects of CD on c-Myc, HIF-1α, and VEGF proteins. (**A**) The protein expressions of c-Myc, HIF-1α and VEGF. GAPDH was used as an internal control. (**B**–**D**) Quantification results of each protein. Bar graphs show normalized values as percentages relative to the control. Data are expressed as mean ± SD of three to four independent experiments. * *p* < 0.05, ** *p* < 0.01, *** *p* < 0.001 as compared to the control group (0 µM). (**E**) The protein interaction map.

This data suggests that CD inhibits the expression of proteins involved in multiple oncogenic pathways, including the EGFR, Akt, mTOR-P70S6K1, and MEK-ERK pathways. We assessed levels of downstream targets of EGFR, c-Myc, and HIF-1α and found that after 24 h of treatment, CD significantly suppresses c-Myc and HIF-1α expression in a concentration-dependent manner (Figure 8A–D). Treatment with CD also reduced the expression of vascular endothelial growth factor (VEGF). Figure 8E displays the predicted pathway through which CD induces apoptosis, cell cycle arrest, and inhibits proliferation. All of the proteins in this network are listed as having strong interactions in the string database (string-db.org).

### 2.6. Expression Profiling of CD-Triggered Cell Death

**Figure 9:**
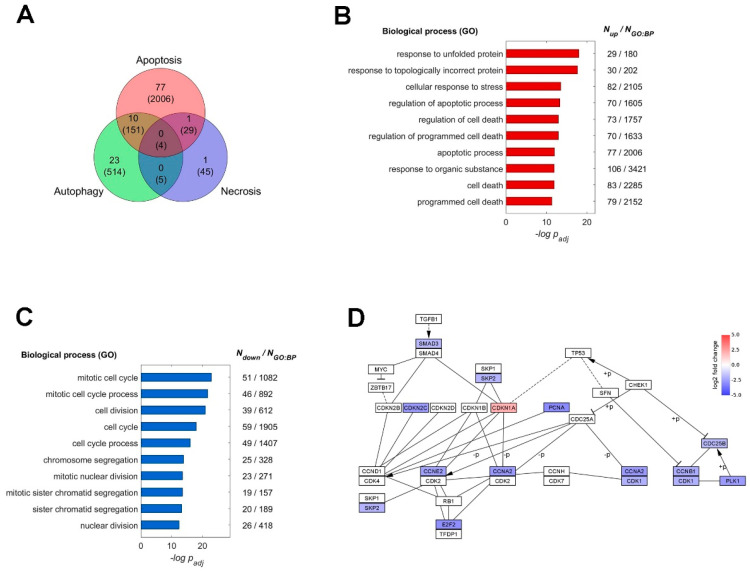
The altered expression profile upon the CD treatment. (**A**) The number of up-regulated genes are depicted in the Venn diagram showing their roles in types of programmed cell death. The intersection areas indicate the number of genes participating in multiple processes. The total number of genes in each category is indicated in parentheses. The top 10 enriched GO biological processes from the up-regulated (**B**) and down-regulated (**C**) genes and with the adjusted *p*-values are plotted on a negative logarithmic scale. The number of up-regulated genes and the total number in each process are also shown on the right of the plot. (**D**) The KEGG pathway of cell cycle progress. Genes with altered expression are color-coded for the log2 fold changes (red: up-regulated; blue: down-regulated; +*p* phosphorylation; −*p* dephosphorylation). The KEGG pathway map was acquired from Kyoto Encyclopedia of Genes and Genomes (https://www.genome.jp/kegg/) and the plot was performed by Cytoscape.

As certain morphological signs of apoptosis were observed after treatment of CD on cultured T24 cells (Figure 2, Figure 3, Figure 4 and Figure 5), we investigated how the cells responded to CD at an earlier time (12 h) through whole-genome expression profiling. The total mapped reads were on average 45,771,919 and 43,699,180 with 95.32% and 95.99% mapping rates for CD treated cells and the control samples, respectively. The threshold to the absolute confect score was set at 0.77, which resulted in a list of 485 significantly expressed genes, including 308 up-regulated and 177 down-regulated ones (Table S1 (Supplementary Material)). Within those up-regulated ones, 77 genes involved in apoptosis and 23 in autophagy were found (Figure 9A). To further determine the detailed responses after CD treatment, functional enrichment analysis was performed on g-Profiler using terms of biological process in the Gene Ontology database (http://geneontology.org/) [16] As shown in Figure 9B, up-regulated genes were involved in the biological processes in response to cell stress, protein unfolding, and organic substances stimulus. In addition, genes involved in cell death and apoptosis were also up-regulated (Figure 9B). On the other hand, down-regulated genes were mainly playing roles in regulating the mitotic cell cycle, such as cell division, nuclear division, chromatid segregation, and so on (Figure 9C). Interestingly, when looking into the signaling pathway of the cell cycle, we noticed an overall suppression of cyclins (CCNE2, CCNA2, CCNB1) and other factors in multiple stages except for the inhibitory CDKN1A (protein p21), which exhibited more than 4-fold up-regulation after the CD treatment (Figure 9D). These features found in the expression profile showed that the T24 cell was responding to stress and under suppression of the cell cycle upon the CD treatment.

## 3. Discussion

BC is the tenth most common cancer worldwide, with approximately 550,000 new cases annually [9,17]. However, little progress has been made in the development of new treatments in recent years. Here, we demonstrate that a clerodane diterpene compound isolated from *P. longifolia* (termed CD) inhibits cell proliferation, stimulates cell cycle arrest, and induces mitochondria-dependent apoptosis. This is associated with inactivation of the EGFR, Akt, mTOR and MEK1/2-ERK1/2 signalling pathways, as well as their downstream effectors c-Myc, HIF-1α and VEGF.

Apoptotic events can be triggered by intrinsic and extrinsic signals [18]. Recent work has focused on inducing mitochondrial dysfunction as a therapy for cancer in recent years [19]. Reduced MMP is an early step in cell death and can be caused by ROS overproduction [19,20]. Our results suggest that CD triggers a loss of MMP and ROS overproduction as well as subsequent release of cytochrome c into the cytosol and activation of caspase-3, PARP-1, and pH2A.X. Thus, our results support the hypothesis that CD induces programmed cell death, at least in part, through mitochondrial-dependent intrinsic apoptosis. On the other hand, the membrane phospholipid containing a high level of polyunsaturated fatty acid is susceptible to ROS attack, which is called lipid peroxidation. ROS-induced lipid peroxidation propagates cytotoxicity and is associated with apoptosis, autophagy, and ferroptosis [21]. RNAseq data indicated that after exposure to CD for 12 h, the MAPK pathway and genes related to ferroptosis were also up-regulated (Figure S1 (Supplementary Material)). Recently, ferroptosis has appeared to be a new approach for manipulating cancer cell death [22]. Research has been shown that activation of the MAPK pathway may sensitize cancer cells to ferroptosis [22]. Moreover, the ferroptotic cell death is driven by iron-dependent lipid peroxidation which accumulates ROS and causes cell death. It raises the higher possibility that CD might induce ferroptosis prior to apoptosis in the T24 BC cell; however, more experiments are needed to clarify. Additionally, recent studies demonstrated that excessive induction of autophagy causes autophagic cell death, overcoming drug resistance in leukaemia and bladder cancer [23,24]. Now we are focusing on the detailed autophagic pathway to determine the connection between ROS, apoptosis, and autophagy, induced by CD treatment in T24 cells.

The effects of CD have been examined on several other cancer cell lines, including oral cancer [5], leukaemia [7], and renal cell carcinoma [8]. The current study is the first to observe the inactivation of the EGFR pathway. In BC, approximately over 50% of tumour specimens presented higher EGFR expression and were associated with recurrent, high-grade, and high-stage patients, as well as having a worse prognosis [25]. It is worth noting that even in the absence of EGFR, the downstream signaling pathways (MAPK and Akt) still activate [14]. Our findings show that CD not only blocked EGFR activation but attenuated two main signaling pathways, as well as preventing HIF-1α, c-Myc, and VEGF expression. In vivo studies have shown that the inhibition of EGFR [26], VEGF [27], and mTOR [28] suppresses bladder tumour growth. These results imply that CD might serve as a good candidate for bladder cancer treatment. Further studies of the specific molecular mechanisms that are involved in CD-induced anti-cancer activity in BC are required.

In conclusion, the present study demonstrates that CD inhibits cell proliferation and induces mitochondrial-dependent apoptosis via an EGFR-mediated signalling network.

## 4. Experimental Procedures

### 4.1. Plant Authentication and Extraction

The leaves of *P. longifolia* were collected locally from Kaohsiung, Taiwan in the summer of 2013 and were verified by Prof. Yi-Chen Chia. The extraction of 16-hydroxycleroda-3,13-dien-15,16-olide was performed as described previously, and the corresponding NMR data is shown by Liu et al. [8].

### 4.2. Cell Culture and Reagents

The human BC cell line T24 was obtained from the Bioresource Collection and Research Centre (Hsin Chu, Taiwan). T24 cells were cultured in McCoy’s 5A medium (Sigma-Aldrich, St. Louis, MO, USA), supplemented with 10% fetal bovine serum (Gibco^TM^, Thermo Fisher Scientific Inc., Waltha, MA, USA) and 1% penicillin-streptomycin (Invitrogen^TM^, Thermo Fisher Scientific Inc., Waltha, MA, USA) at 37 °C with 5% CO_2_.

### 4.3. Morphology Observation and Cell Viability Assay

The morphological changes were observed using a phase-contrast microscope (Leica, Wetzlar, Germany) at 100× magnification. Cell viabilities were measured using a 3-(4,5-dimethylthiazol-2-yl)-2,5-diphenyltetrazolium bromide (MTT, BIO BASIC Inc., Markham, ON, Canda. #T0793) assay. Cells (7500 cells per well) were cultured onto 96-well microplate overnight and were treated with CD (0, 10, 20, 30, and 40 µM) for 24, 48, and 72 h. After treatment, MTT dye (0.5 mg/mL) was added for 2.5 h. The medium was discarded and 100 μL dimethyl sulfoxide was added to extract the MTT dye. The optical density was detected at 570 nm using a microplate reader (Bio-Tek Instruments Inc, Winooski, VT, USA). The survival rates (%) were normalised to the vehicle-treated control group (0 µM).

### 4.4. Acridine Orange and Ethidium Bromide (AO/EB) Double Staining

A dual fluorescent staining solution (3 μL) containing 100 μg/mL AO (Molecular probes^TM^, Thermo Fisher Scientific Inc., Waltha, MA, USA, #A1301) and 100 μg/mL EB (Sigma, #E1510) was added to treated cell suspensions (97 μL) and added to a slide and coverslipped. Cell morphology was examined using a fluorescent microscope with cooled-CCD (NIKON, Tokyo, Japan) at 200× and 400× magnification. AO permeates cells, resulting in cell nuclei fluorescing green, while EB is only taken up by cells with disruptions to cell membrane integrity [29]. Cells that die from necrosis have a structurally normal orange nucleus.

### 4.5. Flow Cytometry Analysis for Annexin V- Propidium Iodide (PI), Reactive Oxygen Species (ROS) and Mitochondrial Membrane Potential ΔΨM (MMP)

T24 cells were plated onto 35 mm dishes overnight and treated with the indicated concentrations of CD for 24 h. Apoptosis was examined using an annexin V- PI detection kit (BioLegends Inc., San Diego, CA, USA. #640914). ROS were measured via incubation of cells with 2’, 7’-dichlorodihydrofluorescein diacetate (DCFH-DA) staining dye (10 μM, AAT Bioquest Inc., Sunnyvale, CA, USA. #15204) for 30 min at 37 °C. MMP was measured after incubation with rhodamine123 (Rh123, 10 μg/mL, Invitrogen^TM^, #R302) and/or 5,5′,6,6′-tetrachloro-1,1′,3,3′-tetraethylbenzimidazolylcarbocyanine iodide (JC-1, 2.5 μM, Molecular Probes^TM^, #T3168) for 30 min at 37 °C. After staining, cells were collected and evaluated using a flow cytometer (Epics XL, Beckman Coulter Inc., Brea, CA, USA) and the results were analysed using Expo32 ADC analysis software (Beckman Coulter). For JC-1, fluorescence was detected at 540 nm and 590 nm wavelengths. The differences in the ratio of intensities between the test wavelengths of (540 nm/590 nm) reflect changes in the MMP of the CD-treated cells.

### 4.6. Fluorescence Staining for MMP and Mitochondrial ROS

T24 cells were treated with different concentrations of CD for 24 h. After treatment, cells were incubated with Rh123 (Invitrogen^TM^, 10 µg/mL) and JC-1 (Molecular probes^TM^, 2.5 µM) for 30 min at 37 °C, respectively. For the detection of mitochondrial superoxide, MitoSOX^TM^ red indicator (2.5 µM, Molecular probes^TM^, #M36008) was performed on the T24 cells for 10 min at 37 °C. Staining was visualised using a cooled-CCD (NIKON, Japan) at 200× and 400× magnifications.

### 4.7. Cell Cycle Analysis

Cells (3 × 10^5^ cells per well) were serum-starved for 24 h prior to incubation with different concentrations of CD. After 24 h of treatment, cells were collected and washed with ice-cold PBS and fixed overnight with 70% ethanol at −20 °C. Cells were then centrifuged at 2000× *g* for 10 min, washed with ice-cold PBS and incubated with the staining buffer (0.2 mg/mL RNase A, 20 μg/mL PI, and 0.1% Triton X-100) for 30 min at 37 °C in the dark. Stained cells were measured using a flow cytometer (Beckman Coulter) and the results were analysed using the Expo32 ADC analysis software (Beckman Coulter).

### 4.8. Western Blot Analysis

Cells were collected and lysed in lysis buffer [150 mM NaCl, 50 mM Tris-HCl (pH 7.2), 0.15% Triton X-100, 2 mM MgCl_2_, 1 mM sodium fluoride, 1 mM sodium orthovanadate, 1 mM glycerophosphate, 2.5 mM sodium pyrophosphate] containing a protease inhibitor cocktail (Roche, Basel, Switzerland). The cytosolic and membrane fractions were separated using a hypotonic buffer containing 0.28 M sucrose, 20 mM HEPES, 50 mM NaCl, 2 mM EDTA, 1 mM sodium fluoride, and a protease inhibitor cocktail (Roche). Cell lysates were separated using SDS-polyacrylamide gels and transferred to an Immobilon-E membrane (Millipore, Darmstadt, Germany). Membranes were blocked with 5% skim milk or 5% bovine serum albumin (for phosphorylated proteins) in Tris-buffered saline (TBS; 150 mM NaCl, 50 mM Tris-HCl, pH8.2) containing 0.1% Tween-20, for 1 h and were subsequently incubated overnight at 4 °C with the indicated primary antibodies. Membranes were incubated with horseradish peroxidase-conjugated or alkaline phosphatase-conjugated secondary antibodies (1:2000) for 2 h at room temperature. Bands were detected using the WesternBright ECL kit (Advansta Inc., Menlo Park, CA, USA) or the NBT/BCIP substrate buffer (Sigma). Primary antibodies used are listed in Table 1.

### 4.9. RNA Extraction and Expression Profiling

T24 cells were treated with or without CD at the concentration of 40 μM for 12 h and then were purified with an RNA kit (Geneaid Biotech Ltd., Taipei, Taiwan) following the instructions. The RNA samples (about 1 μg) were sent to GENEWIZ (Suzhou, China) for RNA sequence analysis. Two experimental replicates for each condition were performed, and the purified RNA samples were sequenced with NovaSeq^TM^ platform (Illumina^®^, San Diego, CA, USA) for the depth of 50 million paired-end reads. The resulting sequence reads were filtered to remove those of short fragments (<75 bp) or with low sequencing quality (Q < 20). The qualified sequence reads were aligned and quantified using the Hisat2 (v2.0.1) program [30] with its implemented bowtie2. The dispersion and difference of expression levels between the CD-treated cells and the untreated were estimated via Deseq2 package [31] using the raw read count data, where a minimum of 10 counts in total across all samples was set as the criteria for the estimation. The resulting log2 fold changes were further evaluated for the confidence bounds using Topconfects [32] The false discovery rate was set to 0.05 and the step size was set to 0.01 for scoring and ranking of the confidence bounds. For enrichment analysis, a confect score of 0.77 was implemented as a threshold to select the genes on the top of the ranked list. The threshold was determined so that the resulting list contained approximately (yet not more than) 500 genes with significantly altered expression. Up-regulated and down-regulated genes were separated and submitted onto the web based g-Profiler for functional enrichment analysis [16].

### 4.10. Statistical Analysis

Quantitative data are expressed as means ± SD from at least three independent experiments using GraphPad Prism 7.0 software (GraphPad Software, San Diego, CA, USA). One-way analysis of variance with Dunnett’s post-hoc was applied to compare the treated group to the control group. *p*-values of <0.05 were considered statistically significant.

## Figures and Tables

**Table 1:** Antibody list.

Antibody	Dilution	Brand
p27Kip1 (#3698), phospho-70 kDa ribosomal protein S6 kinase 1 (pP70S6K1, Thr389; #9234T), phospho-mitogen activated protein kinase kinase 1/2 (pMEK, Ser 217/221; #9121)	1:1000	Cell Signaling Technology Inc. (Danvers, MA, USA)
Caspase-3 (sc-56053), CDK2 (sc-163), CDK4 (sc-260), cyclin D1 (sc-8396), phospho-mammalian target of rapamycin (pmTOR, ser2448) (sc-293132), phospho-extracellular regulated kinase 1/2 (pERK1/2, Thr202/Tyr204; sc-7383) vascular endothelial growth factor (VEGF) (sc-152)	1:1000 1:500	Santa Cruz Biotechnology, Inc. (Santa Cruz, CA, USA)
Cytochrome oxidase subunit IV isoform 1 (COX IV-1, 11242-1-AP) Glyceraldehyde 3 phosphate dehydrogenase (GAPDH, 60004-1-Ig)	1:1000 1:2000	Proteintech Group Inc. (Rosemont, IL, USA)
Hypoxia inducible factor 1 alpha (HIF-1α, 2015-S)	1:1000	Epitomics, Inc., a brand of Abcam (Burlingame, CA, USA)
Cytochrome c (#45-6100)	1:1000	Thermo Fisher Scientific Inc.
B-cell lymphoma 2 (Bcl-2) (B3170), phospho-histone H2A.X (Ser139) (pH2A.X), ZooMAb^®^ (ZRB05636)	1:1000	Sigma
Poly [ADP-ribose] polymerase 1, (PARP-1, E12-173)	1:500	Enogene Biotech Co., Ltd. (New York, NY, USA)
p21 (GTX100444), p53 (GTX102965), c-Myc (GTX103436)	1:1000	GeneTex (Irvine, SC, USA)
pEGFR (Tyr1173) (ab32578), pAkt1 (Ser473) (ab81283) Akt1 (ab32505), mTOR (ab134903)	1:1000	Abcam Plc. (Cambridge, MA, USA)
P70S6K1 (ARG51221), EGFR (ARG66204)	1:1000	arigo Biolaboratories Corp. (Hsin-Chu, Taiwan)

## Supplementary Materials

The following are available online, Figure S1: The top 7 enriched KEGG pathways upon CD treatment. Table S1: Ranked differential gene expression using confect score.

## Abbreviations

ΔΨM	mitochondrial membrane potential
BC	bladder cancer
CCCP	carbonyl cyanide 3-chlorophenylhydrazone
CD	16-Hydroxycleroda-3,13-dien-15,16-olide
JC-1	(5,5′,6,6′-tetrachloro-1,1′,3,3′-tetraethyl-benzimidazolylcarbocyanine iodide)
MMP	mitochondrial membrane potential
MTT	[3-(4, 5-dimethylthiazole-2-yl)-2, 5-diphenyl tetrazolium bromide]
Rh123	rhodamine 123
ROS	reactive oxygen species
