# Supplementary figures and images for: 16-Hydroxycleroda-3,13-Dien-15,16-Olide Induces Apoptosis in Human Bladder Cancer Cells through Cell Cycle Arrest, Mitochondria ROS Overproduction, and Inactivation of EGFR-Related Signalling Pathways
Source: Molecules. 2020 Aug 30;25(17):3958. doi: 10.3390/molecules25173958 (PMC7504739; doi:10.3390/molecules25173958)

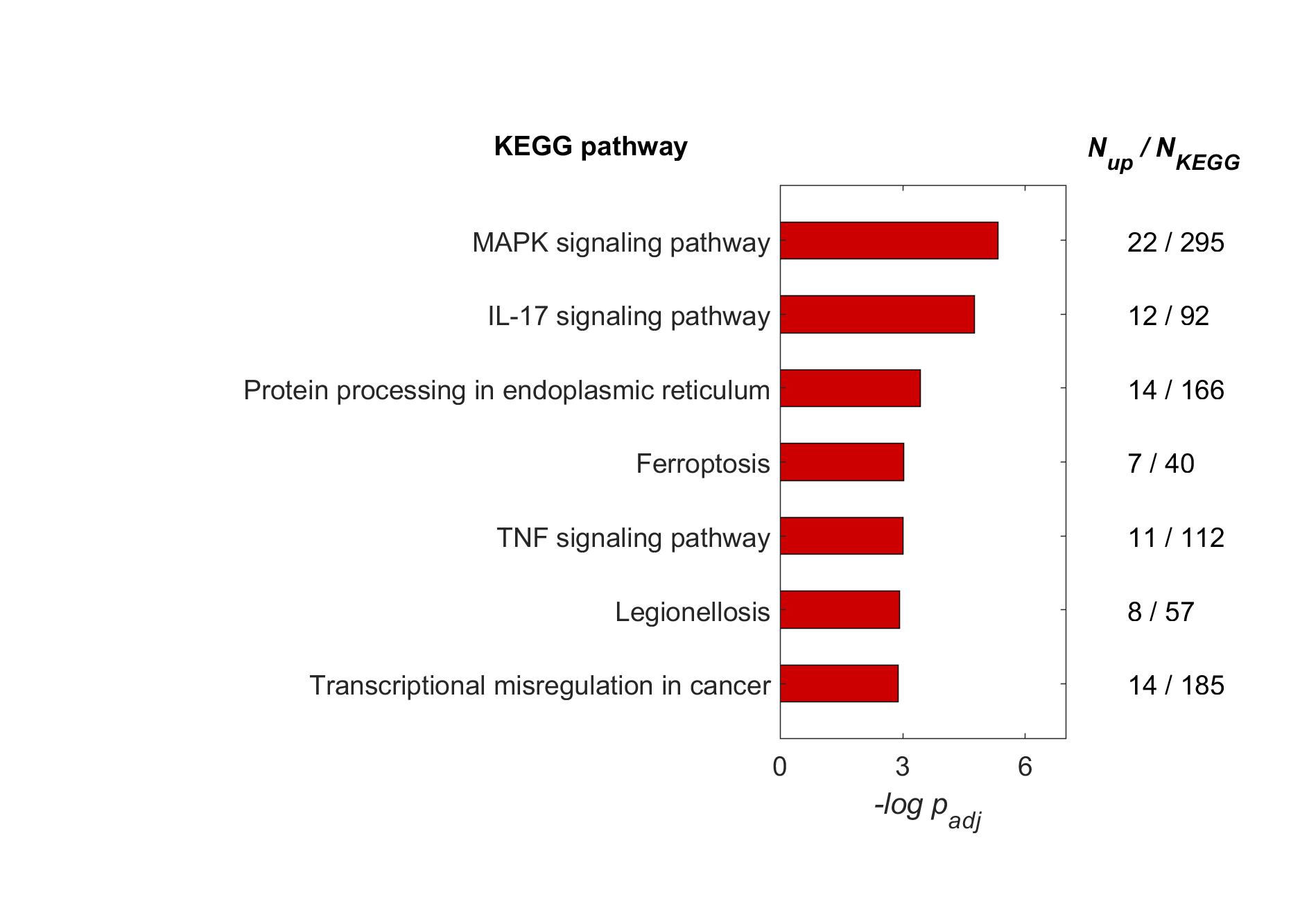

Supplement: Supplementary file 1 [file molecules-25-03958-s001.zip › supplementary 1.jpg]
